# Prevalence of Metabolic Syndrome in Elementary School Children in East of Iran

**DOI:** 10.15171/jcvtr.2015.34

**Published:** 2015-11-29

**Authors:** Mahmoud Zardast, Kokab Namakin, Tayeb Chahkandi, Fatemeh Taheri, Toba Kazemi, Bita Bijari

**Affiliations:** ^1^ Birjand Atherosclerosis and Coronary Artery Diseases Research Center, Birjand University of Medical Sciences, Birjand, Iran; ^2^ Birjand Atherosclerosis and Coronary Artery Diseases Research Center, Department of Cardiology, Birjand University of Medical Sciences, Birjand, Iran

**Keywords:** Metabolic Syndrome, Childhood Obesity, Elementary School Children

## Abstract

***Introduction:*** Metabolic Syndrome (MS) in children and adolescents is becoming a global public health concern. MS tracks into adulthood increasing the risk for type 2 diabetes mellitus and cardiovascular diseases. This study was designed to verify the rate of MS in elementary school students of Birjand, as a representative sample of Iranian children to verify the best preventive measures in this age group.

***Methods:*** This descriptive-analytical, cross-sectional study was performed on 1425 elementary school children through multiple-cluster sampling in 2013. Height, weight, waist circumference and blood pressure of children were measured by standard methods. Blood glucose, triglycerides, cholesterol, High-density lipoprotein-cholesterol (HDL-C) and low-density lipoprotein-cholesterol (LDL-C) levels were also measured after 12 hours fasting. MS was defined according to the Adult Treatment Panel III (ATP-III) based on the National Cholesterol Education Program. Data were analyzed by SPSS using *t* test and chi-square test. Significance level was set at *P* < 0.05.

***Results:*** The prevalence of MS was 5.3% which increased with age. 43.5% of the studied cases had one or more components of the MS. The most common components were hypertension, abdominal obesity, hypertriglyceridemia, low HDL-cholesterol and impaired fasting glucose, respectively. MS prevalence was 0.9% in normal weight, 11.3% in overweight and 36.2% in obese children.

***Conclusion:*** Regarding the high prevalence of MS in elementary school children in our region, screening for obesity is recommended to prevent adulthood complications. Therapeutic lifestyle changes and maintenance of regular physical activity are the most important strategies for preventing childhood obesity.

## Introduction

Metabolic syndrome (MS) is characterized by a set of cardiometabolic risk factors that include abdominal obesity, hypertension, hypertriglyceridemia, hyperglycemia and decreased serum concentration of high-density lipoprotein-cholesterol (HDL-C).^1-3^ MS in children and adolescents is becoming a global public health concern, especially in developing nations. Childhood obesity tracks into adulthood, thus increasing the risk for conditions like the MS, type 2 diabetes mellitus, cardiovascular disease, polycystic ovarian syndrome, hypertension and dyslipidemia later in life.^4,5^ MSis believed to increases the cardiovascular mortality rate by 2.5-fold and the all-cause mortality rate by 1.5-fold.^6,7^ Evaluation of autopsies has shown early atherosclerotic changes in the cardiovascular system in young patients with obesity, hypertension, hypertriglyceridemia, and low HDL-C.^6^ By aging of such patients the rate of cardiovascular diseases increase. Although there are still no definite criteria for the diagnosis of childhood MS, it has been shown that the criteria are mostly similar in childhood and adolescence with overweight and obesity being one of the most important criteria for its diagnoses.^8^

To date, numerous studies have been performed worldwide on different age groups of children suffering from this disorder. The global rate in children is reported as 3.3% (0%-1.2%).^9^ Whereas a prevalence rate of 11.9% has been reported in a recent study from Brazil which reaches up to 29.2% in those with a body mass index (BMI ) >95 percentile.^1^ Even in the United States one in three youth are overweight or obese.^4^

The risk factors of cardiovascular disease are facing a daily increase all around the globe.^10^ Analysis of 144 studies from different countries in 2010, showed that 43 millions of preschool children were overweight or obese, while 35 million of them belonged to developing countries, and 92 million were at the risk of becoming overweight.^11^

As childhood MS is likely to track into adulthood, early identification may help target interventions to minimize future metabolic changes. In order to better assess the extent of preventive and therapeutic strategies required in our region, this study was designed to verify the rate of MS in 6- to 11-year-old students of Birjand’s elementary schools as a representative sample of Iranian children.

## Materials and Methods

### Study Population

This descriptive-analytical, cross-sectional study was carried out in 2013 on 6- to 11-year-old students in Birjand, in the east of Iran.

The study population was calculated as 1296 subjects by the following formula (comparing a ratio with a constant number), considering a prevalence rate of 6.9%^12^ and a CI of 95%.

P= z(1-α/2)^2^ pq/d^2^ =1294.85

The study population was selected through multiple-cluster sampling. Considering the distribution of elementary schools in different districts of Birjand city, at first 10 elementary schools for girls and another 10 for boys were selected. Then, depending on the population of each school and its ratio to the total population of elementary school students, several students were selected from each class.

In this step, 1700 students were selected and a questionnaire plus a consent from were sent to each child’s parents. The parents were demanded to fill out the demographic data questionnaire and sign the informed consent and return them to the school office if they agreed with their kid’s participation in the program; Children with genetic and endocrine disorders, physical condition preventing normal activity, and taking drugs affecting the symptoms of MS were excluded from the study.

In total, 1446 questionnaires were filled out by the parents and returned to the schools.

### Anthropometric Measurements

**Figure 1:**
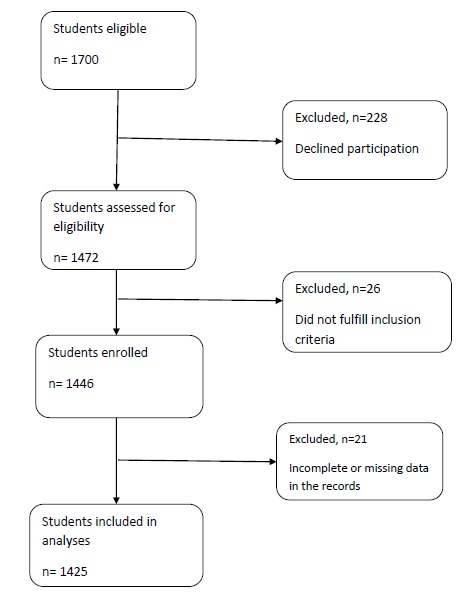
Flow Diagram of the Studied Participants.

In the next step, trained co-workers of the project referred to the aforementioned elementary schools and recorded the weight, height, and waistline data of the participated students by a standard method. At the end, a few cases were excluded due to missing data leaving a final study population of 1425 subjects (Figure 1).

The weighing of the students was done while having light clothes on and being bare-footed by means of the German Seca digital scale allowing for a margin error of 100 g. The height of each student was also measured in a standard manner; allowing for a margin error of 0.5 cm. The waist circumference of each individual was measured while the person was standing and exhaling, by wrapping a measuring strip around the last vertebra and the prominence of iliac, allowing for a margin error of 0.5 cm.

Then, each student’s BMI was calculated. For determining the percentage of overweight and obesity, we used the Center for Disease Control and Prevention (CDC) criteria. So, a BMI between 85% to 95% (for age and sex) was considered as overweight whereas greater than 95 percentile was defined as obese.

### Laboratory Measurements

Blood pressure (BP) was measured twice with a 15 minutes interval by a qualified nurse in the seated position and by using the standard mercury sphygmomanometer (Japan) and an appropriate-sized cuff. The participant’s BP was regarded as the mean value of the two measurements.

Blood samples were taken after overnight fasting and measurement of glucose and lipid levels were done within 1 hour in the laboratory. Fasting plasma glucose (FPG) was measured by the colorimetric enzymatic method using the glucose oxidase enzyme. Total serum cholesterol, and TG, HDL-C and LDL-C concentrations were measured by commercially available enzymatic reagents (ROCHE Kits, Germany with a closed full automated analyzer system of ROCHE COBAS INTEGRA).

MS was detected based on the National Cholesterol Education Program for Adult Treatment Panel III (ATP–III). Subjects with three or more of the following criteria were categorized as having MS: (1) abdominal obesity (waist circumference > age- and sex-specific 90th percentile for this population); (2) elevated BP (systolic and/or diastolic BP > the age-, sex-, and height-specific 90th percentile for systolic and diastolic BP, respectively); (3) low HDL-C level (≤40 mg/dL); (4) elevated serum TGs (>110 mg/dL ); and (5) elevated FPG (>110 mg/dL).

### Statistical Analyses

The collected data were analyzed using SPSS (version 16). The normality distribution of data was examined with Kolmogorov-Smirnov test; indicating a normal distribution. chi-square and *t* test were used whenever appropriate. A *P *< 0.05 was considered as statistically significant.

## Results

### Comparison of Baseline Data by Sex

In total, 1425 subject with the mean age of 9.1 years including 642 (45%) males and 783 (55%) females were examined. No significant difference was observed in the mean height and weight between the two genders. Mean fasting blood sugar was 86.9 ± 8.8 mg/dl, TG: 81.3 ± 37.2 mg/dl and HDL-C: 52.1 ± 10.8 mg/dl. Mean systolic and diastolic BPs were 97.4 ± 19.6 and 55.6±12.8 mm Hg, respectively. Mean waist circumference was 58.4± 8.6 cm, indicating a significant difference based on sex (Table 1).

**Table 1:** Comparison of the Baseline Characteristics of the Study Subjects by Sex

**Variable**	**Boys (n = 642)** **Mean ± SD**	**Girls (n = 783)** **Mean ± SD**	*** P *** ** Value**
Age (y)	9.10 ± 1.43	9.11 ± 1.43	0.84
Weight (kg)	132.39 ± 8.36	132.21 ± 8.88	0.69
Height (cm)	32.20 ± 9.03	29.08 ± 8.73	0.01
BMI (kg/m^ 2 ^)	16.92 ± 3.33	16.29 ± 3.18	<0.001
Waist circumference (cm)	59.15 ± 9.34	57.70 ± 7.97	0.001
FBS (mg/dl)	87.90 ± 7.60	86.10 ± 9.70	<0.05
HDL-C (mg/dl)	53.30 ± 11.40	51.10 ± 10.24	<0.05
Tg (mg/dl)	78.31 ± 38.82	83.81 ± 38.12	0.003
Systolic Blood Pressure (mm Hg)	99.22 ± 20.10	96.12 ± 19.10	0.002
Diastolic Blood Pressure (mm Hg)	57.3 ± 112.81	54.90 ± 12.84	<.0.05

Abbreviations‏: BMI, body mass index; TG, triglyceride; HDL-C, high-density lipoprotein-cholesterol.

### The Incidence of MS

The overall prevalence of MS was 5.3%; 5.7% in girls and 4.8% in boys, indicating no significant difference between the two genders. The prevalence of MS increased with age (*P *= 0.014).

In general, 53.5% of the subjects were normal whereas 43.5% had at least one abnormal component of MS.

The most prevalent MS component was hypertension followed by abdominal obesity, hypertriglyceridemia, low HDL-C and impaired fasting glucose.

### MS Prevalence by BMI

Based on BMI, the prevalence of MS in normal weight children was 0.9% while it was 11.3% in overweight subjects and 36.2% in the obese group.

### MS Symptoms Based on Sex

When comparing the MS components based on sex, high systolic BP and larger waist circumference had a significantly higher prevalence among boys whereas an impaired glucose test and low HDL had a significantly higher prevalence among girls. However, abnormal serum TG level and high diastolic BP showed no meaningful difference between the two genders (Table 2).

**Table 2:** Comparison of the Prevalence Metabolic Syndrome Symptoms by Sex

**Variable**	**Boys** **n (%)**	**Girls** **n (%)**	**P Value**
Increased waist circumference	140 (22.9)	103 (13.2)	<0.001
High systolic blood pressure	163 (25.4.)	163 (20.8)	0.038
High diastolic blood pressure	86 (13.5)	103 (13.2)	0.08
Hyperglycemia	3 (0.5)	13 (1.6)	0.030
Hypertriglyceridemia	102 (15.9)	144 (17.3)	0.126
Low HDL	74 (11.4)	124 (15.8)	0.013

Abbreviation‏: HDL, high-density lipoprotein.

### Comparison of Anthropometric Data

Using logistic regression analysis, sex (*P *= 0.001), waist circumference >90th percentile and BMI > 95th percentile (*P *< 0.001) were significantly associated with the risk of MS occurrence (Table 3).

**Table 3:** Independent Association of Variables With Metabolic Syndrome (Logistic Regression Analysis)

**Variable**	**B**	**SE**	***P*** ** Value**	**Exp (B)** ^a^	**CI (exp B)**
Sex	0.97	0.99	0.001	2.6	1.5-4.7
Waist circumference >90%	3.74	0.45	<0.001	42.1	17.2-102.7
BMI >95%	1.27	0.31	<0.001	3.6	1.9-6.6
Age	0.10	0.10	0.34	1.1	0.9-1.4
Constant	-12.67	1.31	<0.001	<0.001	

Abbreviation‏: BMI, body mass index. ^a^Adjusted OR.

In addition, a significantly higher risk for MS was observed in subjects with a large waist circumference, a BMI above the 85th percentile and those older than 9 years of age. When comparing the two genders adjusted for waist circumference, a significantly higher rate of MS was seen in girls compared to boys among those with a waist circumference over the 90th percentile (Tables 3 and 4).

**Table 4:** Comparison of MS Symptoms in the Studied Subjects Based on BMI and Central Obesity

**Variable**	**Category**	**Metabolic Syndrome**		
		**n (%)**	**OR (CI 95%)**	**OR (CI 95%) adjusted**
BMI > 85% Percentile^c^	Abnormal	65 (24)	35.81 (18.10-70.84)	31.22 (15.93-61.19)^a^
	Normal L	10 (0.9)		
BMI > 95% Percentile^d^	Abnormal	50 (36.2)	28.47 (16.82-48.21)	28.04 (16.38-48.03)^a^
	Normal L	25 (2)		
Wc> 90% Percentile^e^	Abnormal	68 (29.4)	70.68 (31.91-156.52)	83.95 (34.27-205.68)^a^
	Normal L	7 (0.6)		
Age (y)	<9	22 (3.3)	2.26 (1.36-3.76)	1.23 (0.69-2.19)^b^
	≥9	53 (7.1)		
Sex	Male	31 (4.8)	1.20 (0.74-1.92)	2.41 (1.40-4.18)^b^
	Female	44 (5.7)		

Abbreviations‏: BMI, body mass index; MS, metabolic syndrome; OR: odds ratio. ^a^adjusted by age and sex. ^b^adjusted by waist circumference (wc) 90%. ^c^BMI equal or more than of 85% percentile adjusted by sex and age= abnormal. ^d^BMI equal or more than 95% percentile adjusted by sex and age= abnormal. ^e^WC equal or more than 90% percentile adjusted by sex and age= abnormal.

After adjusting for age, the risk of MS in those with a larger waist and a BMI above the 85th and 95th percentiles was 33.1, 35.73 and 27.09 folds greater than normal individuals, respectively. Yet, the prevalence of MS in those over 9 years of age adjusted by waist circumference was not significantly different from younger children (Table 4).

## Discussion

Recent reports indicate that the prevalence of childhood MS has substantially increased during childhood and adolescence due to the increasing rate of childhood obesity on a global scale.^12,13^ If the occurrence of MS in children and adolescents is identified early, risk stratification of future cardiovascular events can be performed.^1^

In this study the prevalence of MS was 5.3% according to the ATP-III criteria. In a study conducted in eight European countries, including 18 745 children the 2.0 to 10.9 years MS prevalence was 5.5% by using reference standards.^14^ Considering the MS prevalence rate in our region, in a study performed in Turkey it was reported as 6.3% in 7-15-year-old children based on the International Diabetes Federation (IDF) criteria.^13^ The same figure was 3% among Qatari school children^15^ and 16.5% to 1.8% among school-aged children in Pakistan based on various definitions.^16^ Prevalence rate of MS is very much dependent on the various definitions offered. In a study conducted in elementary school children of China in 2010 the MS prevalence was reported as 6.6% using the De Ferranti definition.^17^ This figure was 8.9% in 8 to 9-year-old Brazilian children by adopting specific criteria for age.^1^

In a study by Esmaillzadeh et al conducted in Tehran, Iran an MS prevalence of 10.1% among adolescents aged 10–19 years (10.3% in boys and 9.9% in girls) was reported.^18^ In Kelishadi et al study the prevalence of MS was reported as 14.1%.^19^ Yet due to the lack of a universal definition for this syndrome it is difficult to draw a single conclusion.

In the present study, when comparing the two genders adjusted for waist circumference, a significantly higher rate of MS was seen in girls compared to boys among those with a waist circumference over the 90th percentile several other studies have reported a significant difference in the rate of MS between boys and girls with a higher rate among boys.^12,20^ Other studies have reported a slightly higher prevalence of MS in females compared to males.^13,19,21^ However, no meaningful difference in the prevalence of MS based on sex was to other studies from Iran.^18,22^

In the current study, MS was more common in obese children with a prevalence of 0.9% in normal-weighed students, 11.3% in the overweight and 36.2% in the obese group. Obesity, particularly in the central (abdominal) region, has been introduced as a key factor in MS. Childhood obesity seems to play a major role in the high prevalence of pediatric MS in developing countries including Iran. In addition, rapidly changing dietary practices accompanied by an increasingly sedentary lifestyle have contributed greatly to promoting weight gain in recent years. Moreover, obese children are intended to have adult obesity. The findings of our study revealed a prevalence of 9.6%, 9.2%, and 15.7% for overweight, obesity, and central obesity among Birjand’s elementary schools’ students, respectively.

Another study in Birjand’s elementary students in 2002 reported overweight and obesity as 2.2% and 1.2%, respectively.^21^ Overweight was reported in two other studies as 5.2% and 6.1% in 11-15 and 15-18 year-old children, respectively whereas obesity was 2.1%. Comparison the findings of our recent study and those of previous ones indicate a remarkable rise in the prevalence of obesity in Birjand students Similarly, the prevalence of obesity in the United States has increased almost by 50% among adults and by 30% in children in the past 20 years.^23^

In the present study the prevalence of MS in obese children was 36.2% which is higher than the Chinese,^24^ Egyptian^25^ and Turkish children,^13^ but less than three other studies from Iran^26,27^ and Turkey.^28^

Overall, in this study 46.5% of children had at least one of the MS components while the most prevalent MS component was hypertension followed by abdominal obesity, hypertriglyceridemia and low HDL-C. According to Bradshaw et al study,^29^ 38% and 22% of the students without MS had one or two components of the syndrome. Furthermore, 29% of these individuals had low HDL-C, 21% had abdominal obesity and 63% were resistant to insulin, indicating a high percentage of young individuals with high probability of future worsening in cardiometabolic risks. Furthermore, in the latter study the variables of greatest frequency were low HDL-C, abdominal obesity, hypertriglyceridemia and high BP, with a prevalence of 92%, 85%, 77% and 46%, respectively. They concluded that in obese children and adolescents, high BP tends to be more prevalent than lipid abnormalities, similar to our findings.

Taken together, effective health awareness educational programs for children should be immediately put into practice in such nations, despite the many challenges ahead.

Given all the research performed so far, MS and childhood obesity should better be diagnosed early in life, when changes in lifestyle can lower future complications. Schools seem to be the key to achieving this goal by encouraging physical activity besides healthy dietary habits such as increasing more fiber intake and less junk foods and saturated fat.

Eventually, further research on a larger population is needed to identify risk factors in obese children. The longitudinal model design from childhood to adulthood is required to clarify the complexity of MS risk factors while periodic studies are necessary to compare the upcoming changes in the prevalence of obesity in children.

## Limitations of study

The strong points of this study were the large sample size and the efforts made to represent a reliable sample for our region. Yet, due to the cross sectional design certain limitations can be mentioned. The temporal causal relationships could not be measured. We also did not evaluate the children’s life style, physical activity level or family history for cardiovascular disease. Nevertheless, the number of BP measurements is a crucial factor influencing the prevalence of MS which may be decreased upon repeated visits.

## Conclusion

In the present study MS showed a 5.3% total prevalence among school-aged children with a higher rate in those with a BMI >95 percentile. The most prevalent MS component was hypertension followed by abdominal obesity, hypertriglyceridemia, low HDL-C and impaired fasting glucose.

Regarding the high prevalence of MS in elementary school children, screening for obesity is highly recommended. Treatment of these disorders should be focused on changing lifestyle habits, as a child cannot change his or her pubertal progression, ethnicity, or family history.

## Acknowledgments

The authors would like to thank the Vice Chancellor for Research of Birjand University of Medical Sciences for supporting the study financially. Also we appreciate the cooperation of primary schools’ principles and students who participated in this project. We are grateful to Mr. H. Nasrabadi and other executive co-workers who kindly cooperated in the data collection process. The present study was derived from the proposal number 610 approved by the Vice Chancellor for Research of BUMS.

## Ethical issues

The present study protocol was approved by the Ethical Committee of Birjand University of Medical Sciences (BUMS). An informed consent was obtained from the parent/guardian of each participant prior to study entrance.

## Competing interests

The authors had no competing interest to declare.
